# *Salmonella* food-poisoning outbreak linked to the National School Nutrition Programme, North West province, South Africa

**DOI:** 10.4102/sajid.v34i1.124

**Published:** 2019-12-04

**Authors:** Thejane W. Motladiile, John M. Tumbo, Adrien Malumba, Bolaji Adeoti, Nozizwe J. Masekwane, Oleteng M.R. Mokate, Otsile C. Sebekedi

**Affiliations:** 1 National Institute of Communicable Diseases (NICD), Johannesburg, South Africa; 2 Division of Public Health, Surveillance and Response, Provincial Department of Health, Mahikeng, South Africa; 3 North West Department of Health, Bojanala District Health Services, Rustenburg, South Africa; 4 Department of Family Medicine, Sefako Makgatho Health Sciences University, Pretoria, South Africa; 5 Bojanala District Municipal Health and Environmental Services, Rustenburg. South Africa; 6 Division of Communicable Disease Control, North West Provincial Department of Health, Mahikeng, South Africa; 7 Division of Health Programmes, North West Provincial Department of Health, Mahikeng, South Africa

**Keywords:** *Salmonella*, food poisoning, samp, outbreak, response, public school

## Abstract

**Background:**

*Salmonella enterica*, with more than 2500 diverse typhoidal and non-typhoidal serotypes (NTS), are foodborne bacterial pathogens of global public health concern. NTS are the most commonly reported causes of foodborne acute gastroenteritis (AGE). Contaminated food products (meat, poultry, eggs and dairy) have been reported to be a source of more than 95% NTS infections. An outbreak of food poisoning occurred among schoolchildren exposed to food provided by the government-sponsored National School Nutrition Programme (NSNP) at a local public primary day school in North West province, South Africa. An epidemiological, environmental and microbiological investigation was conducted to establish the existence and extent of the outbreak, identify the source(s) and causative agent(s) and institute appropriate control and preventive measures.

**Methods:**

An epidemiological investigation was conducted, including a review of the clinical records of the persons exposed, laboratory testing of the pathological specimens collected, environmental testing of the food samples and food preparation areas.

**Results:**

A total of 164 children developed clinical symptoms of AGE following ingestion of processed maize meal, beans and vegetables. *Salmonella enterica* serovar Heidelberg (*S.* Heidelberg) was the causative agent for this AGE outbreak in 92.0% of the cases. The median duration of illness was 2 days with a case fatality rate (CFR) of 0.6%. The main food product that was contaminated was samp (processed maize meal) that had been poorly stored and prepared (53.4%).

**Conclusion:**

A timeously-initiated epidemiological, environmental and microbiological an investigation led to the conclusion that the etiologic agent of this outbreak was *S.* Heidelberg, and the most probable food vehicle of transmission was cooked samp served to learners within the NSNP.

## Introduction

*Salmonella enterica,* with more than 2500 diverse typhoidal and non-typhoidal serotypes (NTS),^1,2,3,4^ are foodborne bacterial pathogens of global public health concern.^5,6^ Non-typhoidal serotypes are the most commonly reported causes of foodborne acute gastroenteritis (AGE),^4,7,8^ causing about 93.8 million cases (85.6% foodborne) and 155 000 deaths globally per annum; both among children younger than 5 years old and those older than 5 years in the general population.^1,7,8,9,10,11,12^ Nearly 2.6% of the global cas and deaths occur in Africa.^10^ About 60% – 80% of all human *Salmonella* infections (salmonellosis) occur sporadically all over the population of the United States (US).^8^ Despite that, clusters of large outbreaks in places of mass feeding have also been reported recently.^4,8,13^

Contaminated food products (meat, poultry, eggs and dairy) have been reported to be a source of more than 95% of NTS infections.^4,12^ Clinical symptoms include diarrhoea, abdominal cramps, fever, vomiting, nausea and headache, which develop in 12 h – 72 h and last 3–7 days.^6,14^ However, up to 5% of the cases may develop invasive extraintestinal bacteraemia and focal systemic infections requiring effective intravenous (IV) antibiotic therapy or result in death.^9,11^ An estimate of the global and regional burden of invasive NTS (iNTS) infections is still needed in order to inform and stimulate efforts to prevent and manage the illness.^15^

On the early morning of 25 October, a local district hospital notified the North West (NW) Department of Health (DoH) sub-district communicable disease control (CDC) coordinator about an unexpected cluster of nearly 100 learners suffering from AGE. The patients had developed symptoms within 24 hours of having one of the routine brunch meals served by the government-sponsored National School Nutrition Programme (NSNP) at a local public primary day school. The brunch meal was served to entitled school learners (270 out of 301) with parental consent to eat at school on 24 October at about 10:00. The NSNP usually provides dishes of five brunch meal options containing protein, starch and fresh vegetables or fruits per week, with an aim to enhance learning capacity, attendance and punctuality of the learners.^16,17^ Existence of an outbreak was established from an unusual rise in the number of linked school learner cases notified on one day, which exceeded the sub-district’s preceding 9 months’ surveillance threshold by 10 fold.

Upon manifestation of clinical symptoms, most of the cases sought medical help at the local district hospital. On 24 October, the index case presented at the hospital with fever 14 hours after the meal, which was followed by the second case with AGE illness an hour later. Twenty-five additional cases with similar symptoms then followed in rapid succession just before midnight. By the early morning of 25 October, the local hospital casualty and emergency unit received nearly 100 cases. Clinical grading of cases was done followed by appropriate management. Mild cases were empirically treated with cotrimoxazole, antipyretics, spasmolytics and oral rehydration solution (ORS). Severe cases were admitted and treated with IV fluid infusion, cephalosporin and anticonvulsants. Some of the cases had to be stabilised, interviewed and transferred to two nearby public and private health facilities because of limited inpatient bed space.

In response to the outbreak that had attracted media attention,^18^ members of the local multidisciplinary outbreak response team (ORT) and Municipal Health and Environmental Service (MHES) were convened rapidly to initiate an investigation together with the Department of Education (DoE). The National Institute for Communicable Diseases (NICD) was invited by the NW-DoH to strengthen the ORT on 27 October, following initial laboratory test confirmation of a few cases. The aim of the intervention was to conduct epidemiological, environmental and microbiological investigations to establish the existence, describe the extent of the outbreak, identify the vehicle(s) and/or source(s) plus causative agent(s) of infection, and institute appropriate control and preventive measures, whilst preventing similar outbreaks in future.

## Methods

An epidemiological investigation was conducted on persons exposed to the suspected poisoned food and included a review of clinical records, laboratory testing of collected pathological specimens, and environmental testing of the food samples and food preparation areas.

### Descriptive epidemiologic investigation

Medical records including laboratory test results were reviewed, duplicate case records resulting from follow-up visits were removed and descriptive analysis was conducted to characterise the outbreak cases in terms of the three standard epidemiological parameters – person, place and time.^19^ Based on our initial investigation, we hypothesised that food items prepared and served by the NSNP kitchen were the most likely vehicles for the *Salmonella* serogroup B infection. A working case definition was established and shared with nearby healthcare facilities to enable the detection of as many cases as possible.

### Case definition

A suspected case was defined as any person who: (1) attended school and/or their contacts, (2) had ingested at least one of the food items served at school and (3) fell ill between the 24 and 28 October, with any of an AGE illness symptoms (diarrhoea [≥ three loose stools in 24 hours], vomiting, nausea, abdominal pains and fever [body temperature > 38.5 °C]) within 72 hours. A confirmed outbreak case was defined as any person who fulfilled criteria (1) to (3) and tested positive for the *Salmonella* species serogroup B.^20,21^

### Analytical epidemiologic investigation

We conducted an analytical cohort study of all school learners who had eaten any of the food items served by the school on 24 October regardless of being ill (cases) or not (non-cases), in order to test our initial hypothesis regarding the likely vehicles of infection.^21,22,23^ This study design was feasible as lists of all cases and non-cases were available from both the local hospital and school.^24^ Standard triage questionnaires were administered almost immediately to all the self-presenting cases under clinical observation and their parents by the hospital nursing staff during face-to-face interviews, as of 24–28 October. The questionnaires included items on individual case’s identification, demographics, symptoms, onset of illness, treatment, laboratory test and exposure information.^19^ Non-cases, food-handlers and educators were also interviewed by the MHES team.

### Ethical considerations

This investigation was covered under the NICD outbreak or surveillance ethics approval. Permission to conduct and publish the findings of the investigation was granted by the NW-DoH’s Head of Bojanala district research committee (DRC) and the Provincial Director CDC, and was forthcoming from the Head of provincial research committee (PRC). Patient privacy and confidentiality were ensured.

### Statistical analysis

Data entry and analysis were done using an Excel-based (MS Office, Microsoft Corporation, Redmond WA, USA) line-listing and Epi-Info™ 7 (CDC, Atlanta GA, USA), respectively. The Chi-square (χ^2^) test was employed to compute food-specific attack rates (AR) with their corresponding two-tailed *p*-values, and attributable risks or risk differences (RD) among the exposed and unexposed groups. Risk ratios or relative risks (RR) with a binomial 95% confidence interval (CI) were generated to determine the strength of association between the exposure and illness.^20,21,22^ We assumed statistical significance of an association only when *p* < 0.05 and the 95% CI of the RR did not include a value of 1.^2^

### Environmental investigation

Based on preliminary epidemiologic study findings, the MHES team inspected the school kitchen, dishes, utensils, food preparation procedures, food stores and school premises.^4^ The school provided the menu and food safety policies, as well as records of food temperature monitoring and hygiene training.^21^ Swabs of the kitchen work surfaces, leftover food (samp [processed maize meal] and sugar beans) and water samples from five points (kitchen sink taps 1 and 2, and three outside taps) were collected and sent for laboratory testing. Food-handlers were advised and requested to undergo medical screening.

### Microbiological investigation

A number of samples including 13 stools from symptomatic learners (*n* = 10), asymptomatic food-handlers (*n* = 3), leftover samp and sugar beans including their raw materials (*n* = 4), water samples (*n* = 5) and kitchen facility swabs (*n* = 5) were taken for testing at the Infection Control Laboratory (ICL) of the National Health Laboratory Service. Culture and serotyping of common gastrointestinal bacterial pathogens including *Escherichia coli* type 0157, *Clostridium perfringens, Bacillus cereus, Staphylococcus aureus, Vibrio cholera, Yersinia enterocolitica, Salmonella* spp., *Shigella* spp., *Campylobacter* spp. and *Listeria* species were requested.^24^

## Results

### Descriptive epidemiology

**FIGURE 1:**
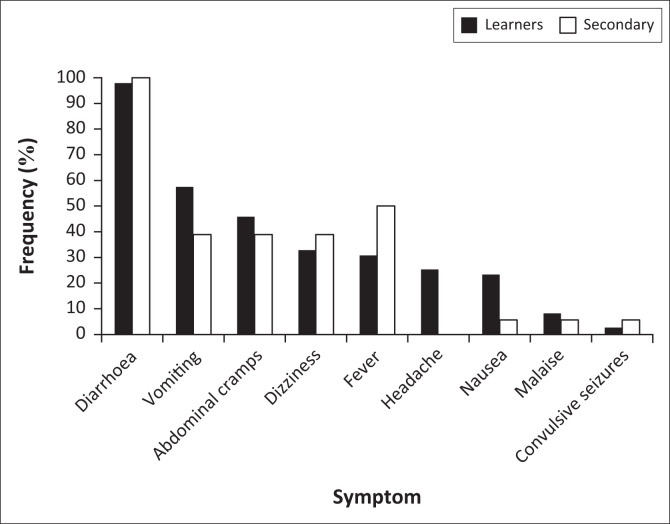
Frequency of symptoms among outbreak learner and secondary cases.

Table 1 shows demographic characteristics of the outbreak cases, as well as the AR. A total of 164 cases were identified – 89% school learners (*n* = 146) plus 11% secondary cases (*n* = 18) that included a deceased individual, giving an overall case fatality rate (CFR) of 0.6%. Of the 299 learners who attended school, a total of 146 met the outbreak case definition, giving overall school and NSNP AR of about 48.5% and 54.0%, respectively. Males and females across Grade 0–9 were almost equally affected by the outbreak. Mean age of the learner cases was estimated to 10.5 ± standard deviation (s.d.) of 2.9 years (range: 6–18), with nearly 90% being children younger than 14 years old (Table 1). Diarrhoea was the most common presentation (97.9% of cases), with convulsive seizures being the least common (approximately 3% of cases) (Figure 1). An overall hospitalisation rate of 5.5% was estimated for severe cases.

**TABLE 1:** Demographic characteristics and attack rates of the outbreak cases – October 2014.

Variables	Characteristic	Attack rates								
		Learner cases			Secondary cases			All cases		
		*N*	%	95% CI	*N*	%	95% CI	*N*	%	95% CI
Race	African	141	96.6	92.2–98.9	18	100	81.5–100.0	159	97.0	93.0–99.0
	Asian	2	1.4	0.2–4.9	-	-	-	2	1.2	0.1–4.3
	Mixed race	2	1.4	0.2–4.9	-	-	-	2	1.2	0.1–4.3
	White	1	0.7	0.0–3.8	-	-	-	1	0.6	0.0–3.4
Gender	Male	74	50.7	42.3–59.0	4	22.2	6.4–47.6	78	47.6	39.7–55.5
	Female	72	49.3	41.0–57.7	13	72.2	46.5–90.3	85	51.8	43.9–59.7
	No record	0	0	0.0–2.4	1	5.6	0.1–27.3	1	0.6	0.0–3.4
Age group	< 5	0	0	0.0–2.5	3	16.7	3.6–41.4	3	1.8	0.4–5.3
	5–9	67	45.9	37.6–54.3	8	44.4	21.5–69.2	75	45.7	37.9–53.7
	10–14	64	43.8	35.6–52.3	4	22.2	6.4–47.6	68	41.5	33.8–49.4
	15–19	15	10.3	5.9–16.4	1	5.6	0.1–27.3	16	9.8	5.7–15.4
	> 20	0	0	0.0–2.5	2	11.1	1.4–34.7	2	1.2	0.1–4.3

CI, confidence interval.

**FIGURE 2:**
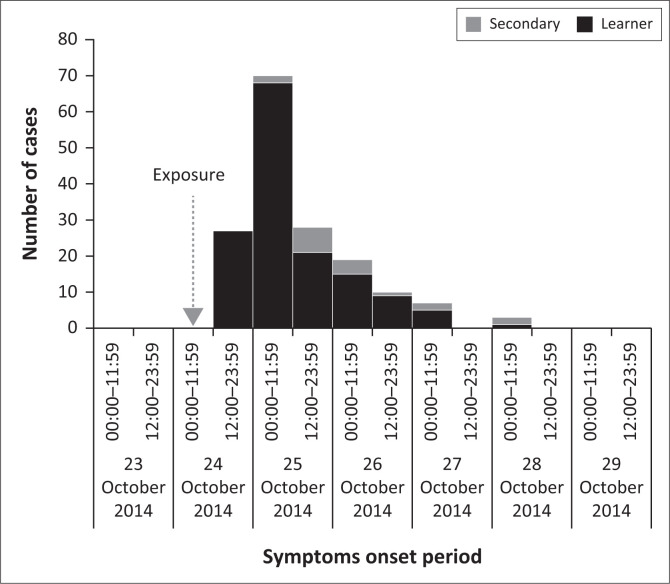
Epidemic curve showing onset of illness among outbreak learner and secondary cases.

The outbreak occurred over a period of 5 days, starting with 27 learner cases on the 24th October, peaked at 98 cases including 9% secondary cases the following day and ending with the last reported three cases on 28 October (Figure 2). The pattern of the epidemic curve is indicative of a point source outbreak starting on 24 October, and then followed by a continuous common source until 28 October (Figure 2).^4,20^

### Analytic epidemiology

Univariate analysis of food items served during the school brunch on 23 and 24 October is shown in Table 2. A significantly high risk of *Salmonella* infection was mostly evident among learners who reported eating only samp (AR = 54.3% [*p* < 0.05]), compared to those eating other foods – sugar beans, rice, soya mince and pasteurised fresh milk (AR = 0.3 to 8.2% [*p* > 0.05]). In addition, the highest attributable risk (RD = 51.2%) was noted for consumption of samp, compared to that of other foods (RD = -2.9% to 5%). Eating samp was significantly associated with an AGE illness outbreak (RR = 2.12, 95% CI 1.83–2.45), whilst eating other foods was protective (RR ± 1.0, 95% CI 0.91–1.13).

**TABLE 2:** Food-specific attack rates and risk ratios for food items consumed at school from 23–24 October.

Date	Meal	Food items	Exposed			Unexposed			RD (%)	Relative risk		*p*-value
			Total	Ill	AR (%)	Total	Ill	AR (%)		RR	(95% CI)	
23 Oct	Brunch	Rice	270	1	0.4	30	1	3.3	−2.9	0.97	0.91–1.04	0.19
		Soya mince	270	1	0.4	30	1	3.3	−2.9	0.97	0.91–1.04	0.19
		Fresh milk	299	1	0.3	1	0	0	0.3	N/A	N/A	0.99
24 Oct	Brunch	Samp	267	145	54.3	32	1	3.1	51.2	2.12	1.83–2.45	< 0.0001
		Sugar beans	268	22	8.2	31	1	3.2	5.0	1.05	0.98–1.13	0.28

N/A, not applicable or undefined; Oct, October; AR, attack rate; RD, risk difference; CI, confidence interval.

* , Statistically significant.

### Environmental health

The MHES reported personal hygiene to be satisfactory, despite some tidiness and maintenance issues, namely, the use of non-liquid hand washing soap, the unavailability of disposable paper towels plus an intercooler system for drinking water and an improper waste water disposable drainage system. There was no evidence of records of food temperature monitoring (during storage, preparation and serving). Records of the training of Grade 3 learners and personnel on personal hygiene and foodborne disease health education, environmental affairs and proper waste management, NSNP monitoring and support visits and guidelines for the management and health surveillance of food-handlers were available. Enquiries about food preparation revealed that samp had been routinely soaked in warm water in a plastic container, pre-cooked and refrigerated overnight for about 16 hours prior to cooking the next morning. On the morning of 24 October, it appeared that bicarbonate of soda was added to the samp to expedite its cooking.

### Microbiology

Of the 13 stool samples cultured for common gastrointestinal bacterial pathogens, 12 (92%) were positive for *Salmonella* serogroup B (10 learner cases and two food-handling personnel) and one (8%) for *Clostridium perfringens*. Further serotyping of all the sample isolates identified *S.* Heidelberg as the causative agent of this outbreak. Only cooked samp showed a matching growth of *S.* Heidelberg, whilst sugar beans, pasteurised milk and water samples all tested negative. Kitchen facility swabs were reported as unsuitable for laboratory testing.

## Discussion

We investigated an outbreak of AGE illness caused by *S.* Heidelberg that affected 48.5% of the primary day school learners including a few epidemiologically-linked secondary cases, following consumption of a common brunch meal prepared and served at school via NSNP. This represents the first large foodborne outbreak linked to NSNP in the North West province of South Africa. Clinical symptoms including self-limiting diarrhoea, vomiting, abdominal cramps and fever, with a median incubation of 15 hours and lasting a median of 3 days,^4,13,25,26^ were consistent with a typical NTS infection.^1,2,6,8,14,26^ Also, the occurrence of invasive extraintestinal bacteraemia leading to convulsive seizures in 3% of the cases corroborated an estimated < 5% global and regional burden of iNTS infections requiring effective IV antibiotic therapy.^1,9^ Based on clinical symptoms, incubation period and illness duration, we concluded that NTS was the causative pathogenic agent of this outbreak.^25,26^ Our conclusion was supported by the microbiological isolation of a common bacterial strain of *S.* Heidelberg in stool samples taken from 10 cases and two asymptomatic food-handlers.

The tracing and collection of fresh leftover plus raw food samples by MHES allowed microbiological genotyping of both cooked plus raw samp and sugar beans. Only cooked samp was confirmed to have a strain of *S.* Heidelberg matching the strain isolated from stool samples taken from the cases and food-handlers. Univariate analysis showed an increased risk (54.3%) of AGE illness to be significantly associated with consumption of dishes containing samp (RR: 2.12, 95% CI 1.83–2.45, *p* < 0.05). Identification of a matching strain of *S.* Heidelberg among the cases, asymptomatic food-handlers, samp prepared and served at school and the epidemiologic association of samp consumption with AGE illness makes an indisputable case for *S.* Heidelberg infection to be in the causal pathway of this outbreak.^25,26^ These findings are thus consistent with our earlier hypothesis that the outbreak was foodborne.^21^ *S.* Heidelberg has been described as a common causative agent for numerous foodborne illness outbreaks in humans.^27^

The epidemic curve representing ingestion of a similar type of meal by a cluster of learners at a single point in time was compatible with a common source outbreak.^4,19^ Direct infection from the consumption of contaminated samp was the most likely source of this outbreak. In addition, it contributed to occurrence of 18 additional secondary cases. About 61% (*n* = 11) of these, including a 4-year-old case fatality, occurred in a household following consumption of samp taken at home after school hours. Possibly, improper storage of the cooked samp could have led to further proliferation of *S.* Heidelberg, causing the additional cases. Though there was no evidence of other modes of spread to the remaining 39% (*n* = 7) secondary cases in the community, person-to-person transmission of *S.* Heidelberg is a possible mode of spread, as described in previous reports.^1,21,26,28^

Studies have highlighted several routes in which samp could have become contaminated by *S.* Heidelberg.^25,26,28^ At first, infected or chronic carrier food-handlers could have transferred the bacteria onto the samp during preparation.^25,26^ According to Von Wissmann et al.,^21^ shedding of pathogens in the prodromal phase of infection and asymptomatic excretion of salmonellae, have been well-documented. Secondly, other possible means of contamination could include pre-cooking and overnight pre-soaking of samp, and the alleged addition of bicarbonate of soda during preparation. Sodium bicarbonate is generally well tolerated. However, high doses could be toxic and may cause clinical manifestations similar to NTS infection, including irritability, constipation and muscle weakness.^29^ Although the use of multiple and raw produce items have been cited to increase the risk of salmonellosis outbreaks,^26^ we did not find any evidence of their use in this investigation.

These investigative findings have some limitations. The delayed meal history-taking could have led to recall bias. We could not confirm or exclude kitchen and utensil contamination as the earlier environmental swabs taken were unsuitable for laboratory testing, and these could not be repeated during the course of the outbreak. Early preventive and control measures including sick leave for ill learners, medical screening and training of food-handlers, detention of stored NSNP kitchen foodstuffs, distribution of proper hand-wash disinfectants, temporary closure of the kitchen, cleaning plus disinfection of the kitchen facility and the permanent exclusion of samp from the menu, may have all successfully prevented occurrence of further cases.^23^

Environmental health investigation revealed some infringements of food safety, including lack of staff training and an absence of records of food safety concepts according to the hazard analysis and critical control points (HACCP) principles. Hence, adequate training of food-handlers on food safety including proper handwashing, disinfection of workplace surfaces plus equipment, preparation and storage processes, is recommended. Food-handler sensitisation to routine medical screening is critical for preventing contamination of food products and hence the occurrence of similar future outbreaks. Should food-handlers fall ill whilst on duty, they must be encouraged to stop working immediately, and resume work not earlier than 2 days after remission of symptoms.^23^ In addition, protective measures should be communicated orally and in the form of poster displays in food preparation units. Lastly, it is imperative to have periodic environmental health inspections that will inform the immediate implementation of policy recommendations.

## Conclusion

A timeously-initiated epidemiological, environmental and microbiological investigation led to the conclusion that the etiologic agent of this outbreak was *S.* Heidelberg, and the most probable food vehicle of transmission was cooked samp served to learners within the NSNP. Also, the investigation proved valuable for guiding targeted intervention measures to prevent further spread of the outbreak. Regular training plus screening of food-handlers, strict personal hygiene, appropriate food-handling and storage and proper disinfection of environmental surfaces remain crucial to prevent further transmission of *S.* Heidelberg. Intersectoral collaboration, involving the departments of education, health, local government and the academic institutions in this investigation, was critical in controlling an outbreak with potential adverse public health consequences.
